# Development and characterization of microsatellite markers for the tsetse species *Glossina brevipalpis* and preliminary population genetics analyses[Fn FN1]

**DOI:** 10.1051/parasite/2023038

**Published:** 2023-09-15

**Authors:** Fabian Gstöttenmayer, Percy Moyaba, Montse Rodriguez, Fernando C. Mulandane, Hermógenes N. Mucache, Luis Neves, Chantel De Beer, Sophie Ravel, Thierry De Meeûs, Robert L. Mach, Marc J. B. Vreysen, Adly M.M. Abd-Alla

**Affiliations:** 1 Insect Pest Control Laboratory, Joint FAO/IAEA Centre of Nuclear Techniques in Food and Agriculture, Vienna International Centre P.O. Box 100 1400 Vienna Austria; 2 Epidemiology, Parasites and Vectors, Agricultural Research Council-Onderstepoort Veterinary Research 100 Soutpan Road Private Bag X05 Onderstepoort 0110 South Africa; 3 University Eduardo Mondlane, Centro de Biotecnologia Av. de Moçambique Km 1.5 Maputo Mozambique; 4 University of Pretoria, Department of Veterinary Tropical Diseases Private Bag X04 Onderstepoort 0110 South Africa; 5 University of Montpellier, Cirad, IRD, Intertryp Campus International de Baillarguet 34398 Montpellier Cedex 5 France; 6 Institute of Chemical, Environmental, and Bioscience Engineering, Vienna University of Technology Gumpendorfer Straße 1a 1060 Vienna Austria

**Keywords:** *Glossina*, Tsetse, Microsatellites, Population genetics, South Africa, Mozambique

## Abstract

Tsetse flies, the vectors of African trypanosomes are of key medical and economic importance and one of the constraints for the development of Africa. Tsetse fly control is one of the most effective and sustainable strategies used for controlling the disease. Knowledge about population structure and level of gene flow between neighbouring populations of the target vector is of high importance to develop appropriate strategies for implementing effective management programmes. Microsatellites are commonly used to identify population structure and assess dispersal of the target populations and have been developed for several tsetse species but were lacking for *Glossina brevipalpis*. In this study, we screened the genome of *G. brevipalpis* to search for suitable microsatellite markers and nine were found to be efficient enough to distinguish between different tsetse populations. The availability of these novel microsatellite loci will help to better understand the population biology of *G. brevipalpis* and to assess the level of gene flow between different populations. Such information will help with the development of appropriate strategies to implement the sterile insect technique (SIT) in the framework of an area-wide integrated pest management (AW-IPM) approach to manage tsetse populations and ultimately address the trypanosomoses problem in these targeted areas.

## Abstract (fr)

Les mouches tsé-tsé, vecteurs des trypanosomes africains, sont d’une importance médicale et économique majeure et l’une des contraintes pour le développement de l’Afrique. La lutte contre la mouche tsé-tsé est l’une des stratégies les plus efficaces et durables utilisées pour contrôler la maladie. La connaissance de la structure de la population et du niveau de flux de gènes entre les populations voisines du vecteur cible est d’une grande importance pour développer des stratégies appropriées pour la mise en œuvre de programmes de gestion efficaces. Les microsatellites sont couramment utilisés pour identifier la structure de la population et évaluer la dispersion des populations cibles et ont été développés pour plusieurs espèces de glossines mais manquaient pour *Glossina brevipalpis*. Dans cette étude, nous avons criblé le génome de *G. brevipalpis* pour rechercher des marqueurs microsatellites appropriés et neuf ont été trouvés suffisamment efficaces pour faire la distinction entre différentes populations de glossines. La disponibilité de ces nouveaux locus microsatellites aidera à mieux comprendre la biologie des populations de *G. brevipalpis* et à évaluer le niveau de flux de gènes entre différentes populations. Ces informations aideront à l’élaboration de stratégies appropriées pour mettre en œuvre la technique de l’insecte stérile dans le cadre d’une approche de lutte antiparasitaire intégrée à l’échelle de la zone pour gérer les populations de glossines et, en fin de compte, résoudre le problème des trypanosomoses dans les zones concernées.

## Introduction

Tsetse flies (Diptera: Glossinidae) are the only cyclical vectors of two parasitic diseases, human African trypanosomiasis (HAT), or sleeping sickness, and African animal trypanosomosis (AAT), or nagana in livestock [6, 33, 58]. Historically, the disease has put a great burden on the development of the African continent and continues to impact food security, mixed crop livestock agriculture and human health [36]. An estimated 70 million people are at risk of contracting HAT and the economic losses due to AAT are significant [25].

Tsetse flies are distributed in sub-Saharan Africa and are part of the dipteran clade Calyptrate, in the group of blood-feeding Hippoboscidae [69], of which many species are of medical and economic importance [25]. All tsetse fly species belong to the genus *Glossina* Wiedeman, 1830 [39]. They are divided into three subgenera according to the differences of structural complexity of the genitalia, which falls in line with body hair patterns and their habitat preferences: flies of the *fusca* group inhabit the lowland rain forests and the border areas of forests and relic isolated forests, the *palpalis* group is found in lowland rain forest of the coastal region of West Africa and extends to the river systems of the humid savannah and the *morsitans* group is restricted the woodland savannah [36, 62]. *Glossina brevipalpis* belongs to the *fusca* group and is feeding on wild mammals and livestock and therefore represents an important vector of AAT [39]. The predicted geographical distribution of *G. brevipalpis* spans from Ethiopia and Somalia in eastern Africa southwards to Uganda, Kenya, Rwanda, Burundi and Tanzania [70]. Furthermore, it is found throughout southern Africa in Malawi, Zambia, Zimbabwe, Mozambique [44] and up to the southernmost populations in KwaZulu-Natal of South Africa [11].

AAT remains a major constraint for more sustainable and efficient livestock development in 36 countries infested with tsetse flies in sub-Saharan Africa. The affected area is approximately 10 million km^2^ of high agricultural and livestock farming potential [24]. Studies project the annual losses in agricultural production due to AAT to be around 3 billion USD, and that the elimination of this burden by coordinated control programmes would need an investment of 12–15 billion USD in a timeframe of 10–15 years [7, 34]. The lack of vaccines against trypanosomoses, the development of resistance to the available trypanocidal drugs against AAT, and the high cost of treatment with chemotherapeutic drugs and their dangerous side effects in humans [27], make the control of tsetse flies an attractive approach for the suppression and management of trypanosomosis [62]. The evolution of tsetse control methods has changed dramatically over the years: from elimination of game and bush clearing during the early 20th century, to insecticide spraying, dipping tanks and insecticide pour-on during the 1950s and the use of insecticide-impregnated traps and tiny targets in recent years [1, 30, 32, 52, 62]. Another control tactic that has proven to be effective is the sterile insect technique (SIT) [61]. SIT, when used as part of an area-wide integrated pest management (AW-IPM) approach, can be used to suppress and even eliminate targeted tsetse fly populations [62]. The SIT requires mass-rearing of the target species in special facilities and sterilisation of males using ionising radiation, followed by systematic and sequential releases in the target area. A sufficient number of sterile males has to be released, so that they can outcompete wild males for mating with wild females. Consequently, mating of a sterile male with a virgin wild female will result in no offspring, which means that the release of massive amounts of such males can considerably reduce the size of the target population at the next generation [62]. An example for the successful implementation of the SIT was the AW-IPM programme on Unguja Island, Zanzibar from 1994 to 1997, where a population of *Glossina austeni* was eliminated and as a consequence, the transmission of trypanosomes on the island was stopped [61].

For the successful implementation of an AW-IPM, knowledge on the level of gene flow between neighbouring populations is required. A tsetse population that is isolated represents the most ideal situation to apply an eradication strategy as it will avoid remigration of flies into the area [20]. This was the case in the Niayes of Senegal, where microsatellites, mitochondrial Cytochrome c oxidase subunit 1 (COI) markers and morphometrics were used to assess population structure. It was found that the tsetse population in the Niayes of Senegal was isolated from the nearest southern populations and, hence, an eradication strategy was selected and implemented [55]. The situation becomes more complex when the targeted insect population is not isolated. Such situations require a “rolling carpet approach” [31] as was implemented during the successful SIT programme against the New World screwworm fly *Cochliomyia hominivorax* in the United States, Mexico and Central America [71].

There are currently several molecular markers available that can be used to gain insight into the population biology of wild species and to assess the level of gene flow between adjacent populations. Among these, microsatellite markers are advantageous, because they do not require sequencing nor a large amount of DNA and are relatively cheap and simple to use [56].

A study using mitochondrial DNA markers and morphometrics was carried out for *G. brevipalpis* and *G. austeni* populations of South Africa, Eswatini (formerly Swaziland) and Mozambique. Although some limitations came with the used markers, the results indicated the absence of barriers to gene flow between the populations in South Africa and Southern Mozambique [13]. The use of microsatellites might help to refine our vision of the structure of these populations and their dispersal. Microsatellite markers have been developed for tsetse species such as *Glossina fuscipes fuscipes* [2, 5, 51], *Glossina palpalis palpalis* [40], *Glossina pallidipes* [47, 48, 51], *Glossina morsitans morsitans* [3, 51] and *Glossina palpalis gambiensis* [51, 54]. No microsatellite loci have been developed specifically for *G. brevipalpis* so far, and attempts to use microsatellite markers developed for *G. pallidipes* gave only limited data for *G. brevipalpis* [48]. Therefore, the aim of this work was to develop novel microsatellite markers for *G. brevipalpis* and to test their suitability for population genetic studies.

## Materials and methods

### Tsetse fly samples

**Figure 1:**
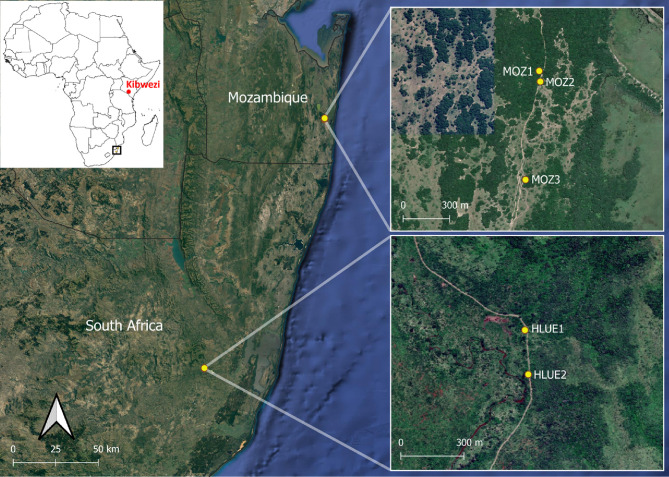
Locations of collected *Glossina brevipalpis* samples in Mozambique and South Africa. Kibwezi Forest (Kenya), the location of collections for Onderstepoort Veterinary Research (OVI) colony is indicated in red.

*Glossina brevipalpis* samples for this study were collected from two field locations in South Africa and three in Mozambique (Table 1) and from a laboratory colony maintained in the Onderstepoort Veterinary Research (South Africa), which originated from flies caught in the Kibwezi Forest in Kenya 41 years ago. Sampling locations, Number of sampled flies, Population label, Number of traps and Geographical coordinates are indicated in Table 1. Sampling locations are furthermore shown in Figure 1.

**Table 1:** Locations and geographical coordinates of tsetse fly *Glossina brevipalpis* samples collected from Mozambique and South Africa and origin of laboratory colony maintained in Onderstepoort Veterinary Research (South Africa).

Location	*N*	Label	No of traps	Latitude	Longitude
1. Hluhluwe-Imfolozi Park 1+ (South Africa)	20	HLUE1	1	−28.09816	32.12231
2. Hluhluwe-Imfolozi Park 2+ (South Africa)	10	HLUE2	1	−28.10031	32.12247
3. Onderstepoort Veterinary Research originated from Kibwezi Forest (Kenya)*	30	OVI (Colony)	0	−2.416917	37.95892
4. Reserva Especial de Maputo 1# (Mozambique)	9	MOZ1	1	−26.59884	32.84528
5. Reserva Especial de Maputo 2# (Mozambique)	7	MOZ2	1	−26.59246	32.84624
6. Reserva Especial de Maputo 3# (Mozambique)	4	MOZ3	3	−26.59177	32.84617

+ Sampled between 15 October and 10 December 2018.

# Sampled between 5 and 7 June 2019.

* Approximate location.

Sampling sites in the two countries were separated by a geographical distance of 162 km. Tsetse flies were collected at Hluhluwe-Imfolozi Park (South Africa) from 15 October 2018 to 10 December 2018 and at Reserva Especial de Maputo (Mozambique) from 5 to 7 June 2019 with odour-baited H traps [37]. Assuming a two-month generation time [41], this represents three tsetse generations between the two sampling campaigns. To enhance the trapping of *G. brevipalpis*, the traps were baited with 1-octen-3-ol and 4-methylphenol at a ratio of 1:8 that were released at 4.4 mg/h and 7.6 mg/h, respectively [38]. These chemicals were dispensed from seven heat-sealed sachets (7 cm × 9 cm) made of low-density polyethylene sleeves (wall thickness 150 microns) placed near the entrance of each trap. A 300 mL brown glass bottle that dispensed acetone through a 6 mm hole in the lid at a rate of ca. 350 mg/h was placed next to the H trap. Flies caught in the traps were guided to a non-return cage that contained a 20% ethanol solution to which an antiseptic, Savlon^®^ (Johnson & Johnson, East London, South Africa) (0.4 mL/L) and formalin (0.4 mL/L) had been added to preserve the sampled flies as well as to prevent ant and spider predation.

Colony flies were obtained from the laboratory colony that has been maintained at the Agricultural Research Council-Onderstepoort Veterinary Research in Pretoria, South Africa for the past 21 years. This colony was originally established from seed material collected in the Kibwezi Forest in Kenya in 1982, with no new genetic material introduced to the colony since then [12]. These colony flies originating from a distant geographic location were included to test the efficiency of the newly developed microsatellites to differentiate between distant and closer field populations.

All collected flies were preserved in absolute ethanol. The samples were prepared for shipment to the Insect Pest Control Laboratory (IPCL) by replacing the ethanol with propylene glycol. Upon arrival, the propylene glycol was replaced by absolute ethanol and stored at −20 °C until DNA extraction.

### Sequence analysis and microsatellite selection

*Glossina brevipalpis* sequence data of the following SRA files (SRR653459, SRR653479, SRR681140 and SRR1174181) available in the SRX220378: Whole Genome Sequencing of tsetse fly project at https://www.ncbi.nlm.nih.gov/sra/ were used. In addition, more sequence data of *G. brevipalpis* were provided by Otto Koekemoer, Agricultural Research Council, Onderstepoort Veterinary Research, South Africa (unpublished data) and used in the analysis. The SRA files were transformed to fastq files using the command “fastq-dump [sra file name]>output file name” in Linux Ubuntu 20.04.4 LTS. The fastq sequence data, consisting of 26209954 reads of 200 bp length were assembled with ABYSS assembler [53] with the command “abyss-pe k=64 np=16 name=sample1_2 in=‘1_2.fastq.gz’”. The .fa files were concatenated to one file with the command “cat file1, file2, filexx>all_file”. The concatenated file was screened for di- and trinucleotide motifs with a minimum of ten repeats using MSATCOMMANDER 1.08 [23] which also enables primer design with the integrated PRIMER3 software. The location of the extracted primers on the *G. brevipalpis* complete genome (https://vectorbase.org/vectorbase/app/downloads/release-59/GbrevipalpisIAEA/fasta/data/, accessed on 8 August 2022) and in coding sequence regions (CDC) was determined using Geneious Prime software, version 2022.2.2. Microsatellites with dinucleotide motifs were prioritised as they showed higher levels of polymorphism compared to trinucleotide motifs in a previous study on tsetse flies [4]. A total of 188 primer pairs were selected based on product size (180–380 bp) and the number of repeats (≥13 repeats), synthesized by Eurofins Genomics (Ebersberg, Germany), and tested for microsatellite amplification with PCR.

### DNA extraction and PCR amplification

Only male tsetse fly samples were used for the microsatellite validation in this study in order to be able to exclude loci on the X chromosome. Samples were rehydrated in distilled water for 5 min after removal of the ethanol and separated into two sample types: whole body and leg samples. Total genomic DNA of *G. brevipalpis* body samples was extracted using DNeasy Blood & Tissue kit (QIAGEN Inc., Redwood City, CA, USA), following the manufacturer’s instructions. DNA of three legs per fly was extracted with a Quick-DNA^TM^ Miniprep Kit (Zymo Research, Irvine, CA, USA). The quantity and quality of extracted DNA was assessed with a Synergy H1 Hybrid Multi-Mode Reader (Agilent Technologies, Inc., Santa Clara, CA, USA).

In the first phase of the study, 188 synthesized primer pairs were tested with DNA extracted from five *G. brevipalpis* flies (whole body samples) to determine primer specificity and the amplicon profile. PCR amplification was carried out in a total reaction volume of 25 μL, with 12.5 μL QIAGEN Taq PCR 2X Master Mix (QIAGEN Inc.), 10 μL nuclease-free H_2_O (Qiagen Inc., CA, USA), 0.2 μM of each primer and 1.5 μL (4 ng) of DNA. All primer sequences can be found in Supplementary Table 1. PCR conditions were 94 °C for 2 min; 35 cycles of 94 °C for 30 s, 58 °C for 30 s, and 72 °C for 1 min; ending with a final extension at 72 °C for 5 min. The PCR amplification was checked on 2% agarose E-gel^TM^ stained with ethidium bromide (Invitrogen, ThermoFisher Scientific, Waltham, MA, USA). Out of 188 tested primers, 170 successfully amplified the expected band sizes, from which 20 were selected to be tested with 10 leg samples from each population to assess heterogeneity of fragment sizes between and within populations. A set of 12 microsatellites (Gb5, Gb28, Gb35, Gb48, Gb50, Gb66, Gb70, Gb72, Gb73, Gb92, Gb158, Gb165) exhibiting polymorphic patterns between populations was selected to be amplified for the final phase of the study, which involved amplification of the microsatellites with fluorescent dyes (6-FAM, HEX, ATTO 550 and ATTO 565). This was done by synthesizing the forward primers linked with the M13 adapter (5′-CACGACGTTGTAAAACGAC-3′). PCR was performed in a total reaction volume of 25 μL, with 12.5 μL Platinum^TM^ II Hot Start PCR 2X Master Mix (ThermoFisher Scientific), 9.6 μL nuclease-free H_2_O (QIAGEN Inc.), 0.016 μM forward primer with M13 adapter, 0.2 μM reverse primer, 0.2 μM M13 adapter labelled with fluorescent dye (6-FAM, HEX, ATTO 550 or ATTO 565) and 1.5 μL of 1:5 diluted DNA (0.75–26 ng/μL). The PCR conditions were as follows: 94 °C for 2 min; 35 cycles at 94 °C for 15 s, 58 °C for 15 s, and 68 °C for 15 s; ending with a final extension at 68 °C for 5 min. PCR products were checked on 4% agarose E-gel^TM^ stained with ethidium bromide (Invitrogen). PCR products were then resolved on an ABI 3500XL Genetic Analyzer (Applied Biosystems, Waltham, MA, USA) with a GeneScan^TM^ 600 LIZ^TM^ internal size standard (ThermoFisher Scientific).

### Quality assessment of *G. brevipalpis* microsatellite markers

Raw data reads were processed and allele calling performed in Genemapper version 6 (ThermoFisher Scientific). Allele calls were transformed into a codominant matrix displaying the microsatellite loci and their respective alleles. The genetic data were formatted for Create v. 1.37 software [10], to convert the datasets into the required formats according to the software used. For Mozambique samples, individual traps were considered subpopulations, except for MOZ3, where samples from three traps, located within 50 m distance, were pooled due to the low number of samples per trap. Each of the two traps from South Africa was considered a subpopulation.

Quality of data was tested with Fstat v. 2.9.4. [28]. Presence of linkage disequilibrium (LD) between each locus pair was checked using *G*-based tests with 10,000 randomisations [29]. Furthermore *p*-values were corrected according to Benjamini and Yekutieli (BY) to assess for the false discovery rate [4] in RStudio v. 2021.09.2 [50]. *F*-statistics, namely Wright’s *F*_IS_ for estimation of deviation from panmixia of genotypic frequencies at local scales (e.g. within subsamples), Wright’s *F*_ST_ for estimation of subdivision, and Wright’s *F*_IT_, a measure of deviation from panmixia at the whole sample scale (which results from the latter two parameters: (1 − *F*_IT_) = (1 − *F*_IS_)(1 − *F*_ST_) [18]), were estimated using Weir and Cockerham’s unbiased estimators [68]. Significant deviation from panmixia and significant subdivision were evaluated with 10,000 permutations of alleles between individuals within subsamples as well as individuals between subsamples, respectively. For the first, the statistic used was simply Weir and Cockerham’s estimator of *F*_IS_ and for the second, the statistic used was the log-likelihood ratio *G* [29]. Confidence intervals were calculated with 5000 bootstraps over all loci.

As departures from Hardy-Weinberg-Equilibrium (HWE) may be caused by a Wahlund effect or genotyping errors such as short allele dominance (SAD), null alleles or stuttering [17], several tests were performed to investigate the influence of these scenarios on HWE. Detection of null alleles, SAD and stuttering was assessed using the strategy described in several papers [14, 16, 17, 19, 41]. The frequency of null alleles was estimated with the EM algorithm [21] using FreeNA. One-sided exact binomial tests were performed in RStudio v. 2021.09.2 [50] to test the goodness of fit of expected null homozygotes and observed missing data (putative observed null homozygotes).

### Genetic differentiation

Measure of genetic differentiation was assessed with Wright’s *F*_ST_, corrected for the presence of null alleles with the excluding null alleles (ENA) method implemented in FreeNA [9], and labelled *F*_ST_FreeNA_. The 95% confidence intervals with 5000 bootstraps over loci were also computed with FreeNA. For these computations, missing data were recoded as homozygotes for null alleles, i.e. 999,999, as recommended [9]. A standardised measure, corrected for the excess of polymorphism, was obtained with *F*_ST_FreeNA_′) = *F*_ST_FreeNA_/*F*_ST_Fmax_, where *F*_ST_max_ was calculated using the software Recodedata [42]. We computed these quantities between each pair of subsamples within the two countries and computed the averages of the means across loci and of the 95% confidence intervals. We also computed *G*_ST_″ = [*n*(*H*_T_ − *H*_S_]/[(*nH*_T_ − *H*_S_)(1 − *H*_S_)] [43], where *H*_T_ and *H*_S_ are total and local genetic diversities [45] and *n* the number of subsamples. According to Wang [64], when the correlation between Nei’s *G*_ST_ and *H*_S_ is negative, *F*_ST_′ (Meirmans) offers a more accurate estimate of subdivision, while *G*_ST_″ performs better otherwise. The correlation between *G*_ST_ and *H*_S_ was measured and tested with a one-sided Spearman’s rank correlation test with rcmdr.

### Effective population sizes (*N*_e_)

Effective population sizes (*N*_e_) – roughly the number of reproducing adults in a population – were estimated for wild tsetse fly samples. We utilised five different methods: linkage disequilibrium [67] corrected for missing data [49] and molecular co-ancestry [46] as computed with NeEstimator v. 2.1. [22]; the heterozygote excess method recently proposed by [15]) *N*_e_ = −(1/2*F*_IS_) − (*F*_IS_/2(1 + *F*_IS_)) the one- and two-locus identity probabilities [59] with ESTIM v. 1.2. [60]; and Wang’s sibship frequency method [65] with the software Colony v. 1.0.6.8 (January 5, 2022) [35]. We then computed the average *N*_e_ across methods, weighted with the number of usable values (i.e., values different from 0 or infinity).

### Genetic relationships between wild populations and the laboratory colony of *G. brevipalpis*

Cavalli-Sforza and Edwards’ chord distances [8] between subpopulations were computed with FreeNA with including null alleles (INA) correction for null alleles [9], which served as input for MEGA11 [57] to construct a Neighbour-Joining tree. To further visualise genetic structure, a Factorial Correspondence Analysis (FCA) was undertaken with GENETIX v. 4.0.5.2. Significance of the 10 first axes was evaluated with the broken stick method [26].

## Results

### Microsatellite development and validation

The MSATCOMMANDER search for di- and trinucleotides resulted in a total of 55,664 motifs and 21,827 pairs of primers. Out of them, 1244 primer pairs could be found in duplicates in the genome and therefore were excluded from the analysis. Combining the unique primer pairs with the motifs produced 20,583 primers pairs, out of them 13,420 primer pairs flanked repeats of dinucleotides and 7163 flanked trinucleotide repeats. Mapping the unique primers (no duplicates) to the *G. brevipalpis* genome indicated that all primers (41,166) mapped to the genome. When mapping the primers to the coding sequence regions (CDS), it showed that 24,609 primers matched to CDS regions, while 16,557 matched to sequences between CDS. Selecting primer pairs that produced PCR product ranging between 180 and 300 nucleotides resulted in 7328 primer pairs. Sorting the primer pairs with the number of motifs with a cut-off ≥ 13 repeats resulted in 253 primer pairs out of which 188 primer pairs with the highest number of motif repeats were synthesized and tested by PCR (Supplementary Table 1). Out of the 188 primer pairs tested by PCR, 170 (90.4%) showed amplification at the expected fragment size. Among all tested primer pairs, 103 (54.7%) showed monomorphic and 67 (35.6%) indicated polymorphic amplicons. From the microsatellites showing polymorphism, 12 (Gb5, Gb28, Gb35, Gb48, Gb50, Gb66, Gb70, Gb72, Gb73, Gb92, Gb158, and Gb165) were selected for population genetics analyses.

### Quality assessment of microsatellite loci

**Figure 2:**
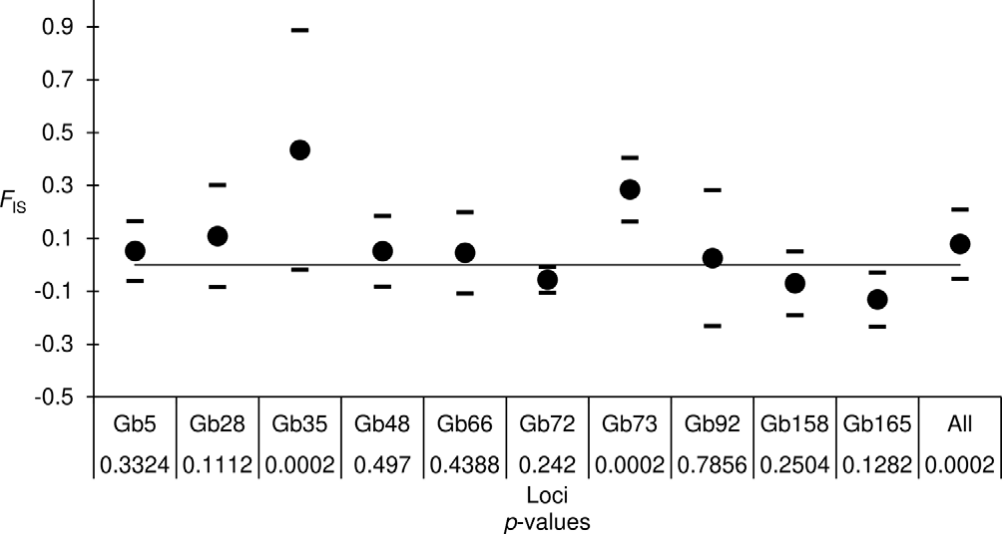
Average homozygosity index (*F*_IS_) by locus with upper and lower limit as calculated with the formulas Li = *F*_IS_ – StdErr × *t* and Ls = *F*_IS_ + StdErr × *t* and over all loci (All) estimated over all subpopulations. The 95% confidence interval for all loci was obtained by jackknife resampling over populations for each locus and by 10,000 bootstraps over loci for the average (All). The two-sided *p*-values obtained while testing for significant deviation from panmixia and the number of observed missing data are also indicated under locus names.

**Supplementary Figure 1:**
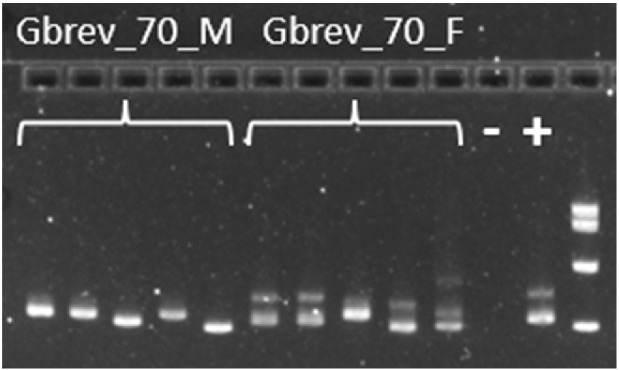
Determination of X-linkage for locus Gb70. PCR products were migrated on 4% agarose gel stained with ethidium bromide. The first five samples are males, the last five females. Template DNA of positive control (+) was derived from 1 female sample. M: FastRuler Low Range DNA Ladder (ThermoFisher) with band size top to bottom are 1 500, 850, 400 and 200 bp.

**Supplementary Figure 2:**
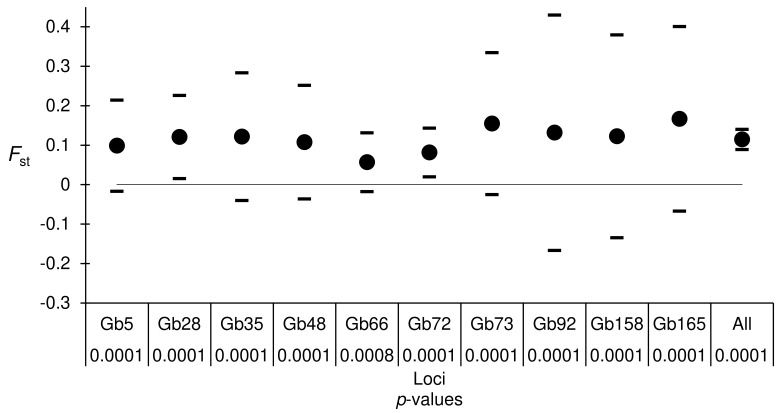
*F*_ST_ values with *p*-values for subdivision of 10 loci across all samples. Dots indicate mean values, dashes show lower and upper limits calculated with the following formula: Li = *F*_ST_ – StdErr × *t* and Ls = *F*_ST_ + StdErr × *t*. Dashes for All loci are the 95% confidence intervals as obtained by jackknife resampling over populations for each locus and by 10,000 bootstraps over loci for the average.

Out of the 12 microsatellites, Gb50 was excluded due to the low success of fragment analysis reads. Gb70 was considered X-linked as it displayed different amplicon patterns with DNA from females comparing to males in preliminary tests (Supplementary Figure 1). Therefore, it was excluded from analysis due to X-linkage. Overall, 10 microsatellites were subjected to the quality control tests (Supplementary Table 2). Primer sequences, microsatellite motifs, number of alleles, allele size range and genetic diversities of each locus are presented in Table 2. The quality parameters, namely *F*_IS_, *F*_ST_, linkage disequilibrium (LD), short allele dominance (SAD), stuttering and presence of null alleles were first assessed for 10 loci on all tested samples. The *G*-based tests for LD between each pair of loci indicated that four locus pairs were in disequilibrium (Gb5xGb66, Gb28xGb72, Gb28xGb92 and Gb35x92); however, none of the pairs gave a significant *p*-value at the BY level. The global analysis over all loci and all samples indicated a significant heterozygote deficit *F*_IS_ = 0.079 in 95% CI [−0.052, 0.2101] (*p*-value = 0.0002), as indicated in Figure 2. Genetic differentiation was significant, with minor variation across loci: *F*_ST_ = 0.115 in 95% CI [0.089, 0.141] (*p*-value = 0.0001) (Supplementary Figure 2).

**Table 2:** The 10 polymorphic microsatellite loci selected for *Glossina brevipalpis* including repeat motif, primer sequence, number of alleles (*N*_A_), allele size range, heterozygosity within subsamples (*H*_s_), total heterozygosity (*H*_t_).

Locus name	Repeat motif	Primer sequence (5’-3’)	*N*_A_	Allele size range (bp)^a^	*H*_s_	*H*_t_
Gb5	AG(21)	F: GTTACTAGTCACTCATAAGCACATG	13	219–247	0.851	0.899
		R: CTCATATATGCTTCTTGTTGGTCC				
Gb28	AG(18)	F: CTACTACCAACTCCAATCAAAGAAG	12	227–281	0.799	0.867
		R: TCTACTCGCTAAGAAGTAAATCCTG				
Gb35	AC(17)	F: TTTCTCCTTAGCCTGCATTAGATAG	10	193–239	0.673	0.783
		R: AAACACACACTAACTGAGAGAGG				
Gb48	AC(16)	F: CTAAACCTCTAGTGAAACAGAAGC	14	229–279	0.809	0.867
		R: CTATAACATAACTCTAACGCTACGG				
Gb66	ATC(16)	F: CAAACCTGTTGCATCTTTGTTTGG	14	202–259	0.861	0.893
		R: CCTCGTCACTACCATCGTC				
Gb72	AC(15)	F: TTGTCACATATATGAGAACGACCTC	14	207–245	0.798	0.841
		R: GAAGTTCACTACAATGTCATCTGC				
Gb73	AC(15)	F: TTTCTGATGTTGGAGAGTGTCTAC	11	228–256	0.764	0.849
		R: CCATTTGATTCACGATAACCAACC				
Gb92	AG(15)	F: GAGAGCGAGAGACAACATTTC	13	278–324	0.755	0.802
		R: TTTCGTTATCTCCCTCTTTCGTTTG				
Gb158	AC(13)	F: CACCTCAGCCTTCATAAATTAACAC	8	187–205	0.744	0.805
		R: GCTAACAAAGAGGAGTATGTAC				
Gb165	AC(13)	F: TTTGGGAGAAACACGTACGAC	5	210–220	0.534	0.604
		R: GTTGTATACTTAAGCAACGCACAC				

a Length including M13 adapter.

**Supplementary Figure 3:**
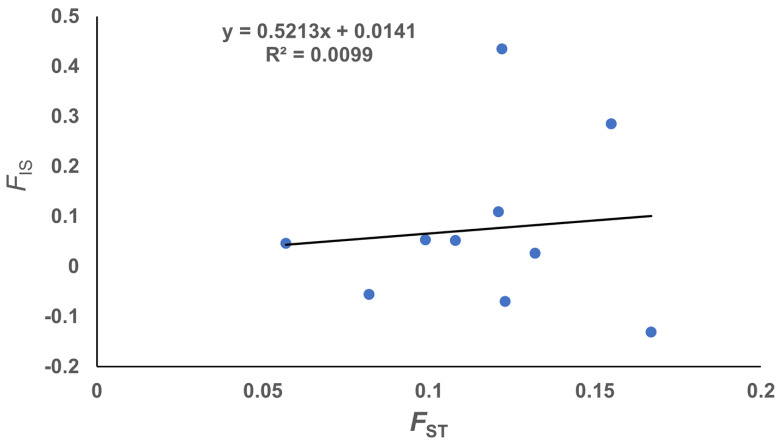
Correlation between *F*_IS_ and *F*_ST_ and between *F*_IS_ and the number of blanks. (A) Correlation between *F*_IS_ and *F*_ST_ for 10 loci across all samples. (B) Correlation between *F*_IS_ and number of blanks for all loci across all samples. (C) Correlation between *F*_IS_ and number of blanks for 4 selected loci (Gb66 [Nb: 1], Gb 5 [Nb: 2], Gb73 [Nb: 5] and Gb35 [Nb: 10]) across all samples.

**Supplementary Figure 4:**
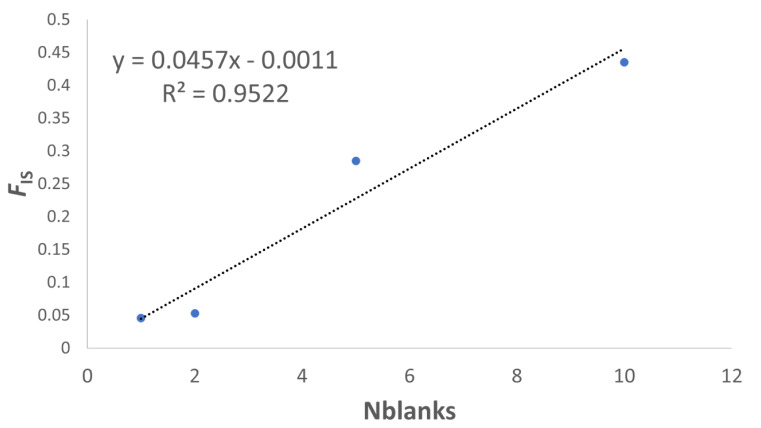
Wright’s *F*_IS_ values *p*-values for panmixia (A) and *F*_ST_ values with *p*-values for subdivision (B) of nine selected loci across all samples. Dots indicate mean values, dashes show lower and upper limits calculated with the following formula: Li = *F*_XY_ – StdErr × *t* and Ls = *F*_XY_ + StdErr × *t*. Dashes for All loci are the 95% confidence intervals as obtained by jackknife resampling over populations for each locus and by 10,000 bootstraps over loci for the average.

The correlation between *F*_IS_ and *F*_ST_ across all loci was not significant (*p*-value: 0.6336) with a trend to be negative (*ρ* = −0.115), indicating that null alleles do not affect *F*_ST_ enough to result in a significant positive correlation with *F*_IS_ (Supplementary Figure 3). The Spearman correlation between the *F*_IS_ and the observed missing genotypes (blanks) for some loci (Gb5, Gb35, Gb66 and Gb73) was significantly positive (*p*-value = 0.04167) and at least 95% of the variance of *F*_IS_ is explained by the number of blank genotypes found across these loci (Supplementary Figure 4). Other loci displayed an excess of missing genotypes. No significant stuttering signature was observed for all tested microsatellites. However, an indication of SAD was detected for the locus Gb48 with a *p*-value of 0.09867 and negative *ρ* (−0.367033) with Spearman’s rank correlation test. The weighted regression correlation test was significant (*p*-value = 0.0456), therefore Gb48 was excluded from further analysis. In conclusion, nine microsatellites (Gb5, Gb28, Gb35, Gb66, Gb72, Gb73, Gb92, Gb158 and Gb165) were retained for further analysis after excluding Gb50 for low success of fragment analyser reads, Gb70 for X-linkage and Gb48 for SAD.

**Supplementary Figure 5:**
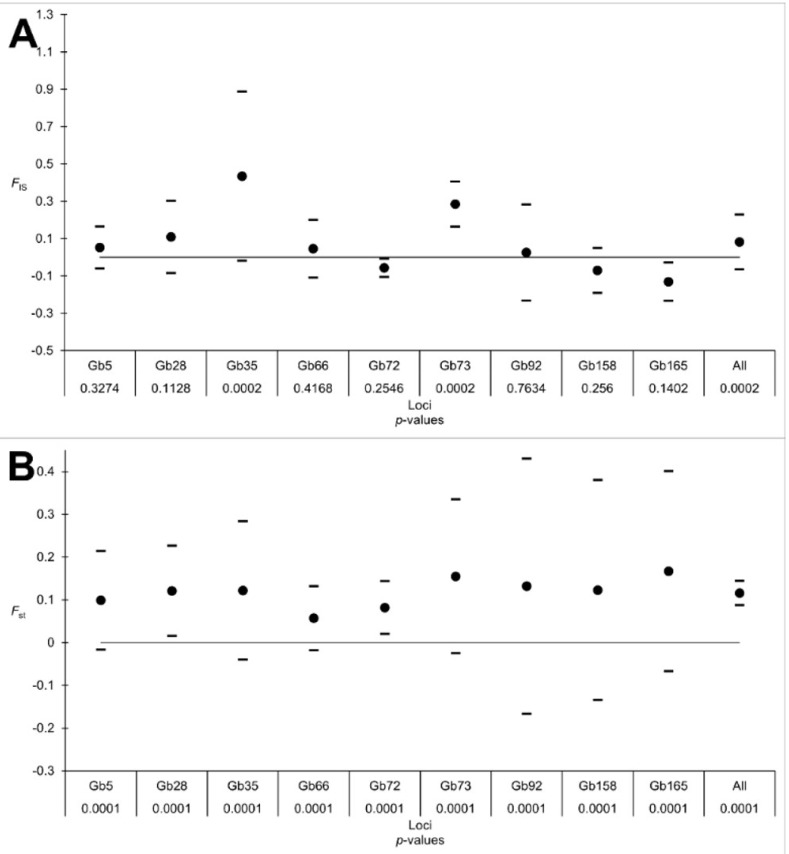
Correlation between *F*_IS_ and *F*_ST_ and between *F*_IS_ and the number of blanks. (A) Correlation between *F*_IS_ and *F*_ST_ for selected 9 loci across all samples. (B) Correlation between *F*_IS_ and number of blanks for selected nine loci across all samples. C: Correlation between *F*_IS_ and number of blanks for four selected loci (Gb5 [Nb: 0], Gb66 [Nb: 1], Gb73 [Nb: 3] and Gb35 [Nb:6]) across all samples.

Analysing the data from all samples with the nine selected loci resulted in a total heterozygosity (*H*_t_) of 0.816. The overall *F*_IS_ decreased to 0.082 (Supplementary Figure 5A). Genetic differentiation remained significant (*p*-value = 0.0001), with a marginal increase of *F*_ST_ to 0.116. Four locus pairs were in significant LD (Gb5xGb66, Gb28xGb72, Gb28xGb92 and Gb35xGb92); again none of these pairs remained significant after BY-correction. The correlation test between *F*_IS_ and *F*_ST_ remained non-significant (*p*-value: 0.6206) (Supplementary Figure 5B).

### Genetic differences between laboratory colony and wild flies from Mozambique and South Africa

To determine the genetic relationship between the *G. brevipalpis* from a colony maintained in culture for 40 years and the field collected flies, we conducted an FCA (individual based) and NJ-tree analysis based on Cavalli-Sforza and Edwards chord distances between subsamples, with INA correction for null alleles.

**Figure 3:**
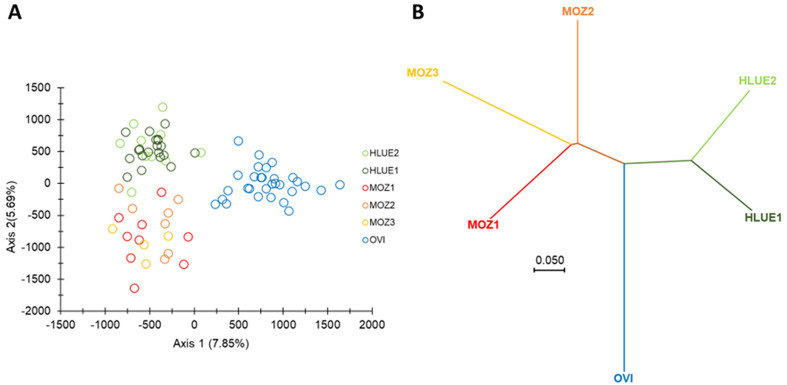
Population structure of *G. brevipalpis*. (A) Factorial Correspondence Analysis of six *G. brevipalpis* field and laboratory colony populations on nine selected loci as computed with GENETIX v. 4.0.5.2. For each axis, percentages of inertia are given. (B) Neighbour-joining tree constructed with MEGA11 based on Cavalli-Sforza and Edward’s chord distances between individuals. Branches are labelled according to the sampling site: HLUE 1 and HLUE 2 (South Africa 1 and 2) – green; OVI (Onderstepoort Veterinary Institute) – blue and MOZ 1, 2 and 3 (Mozambique 1, 2 and 3) – red.

The OVI laboratory colony appeared distant from the other two wild subpopulations (Figure 3A). Regarding the FCA, this differentiation was the main contributor of the first axis of the FCA, while the second axis clearly separated wild subsamples from Mozambique and South Africa, though with less strength (Figure 3B). Furthermore, as seen in the NJ-tree, OVI obviously represents an outgroup.

### Quality assessment of selected microsatellite loci for field samples only

**Supplementary Figure 6:**
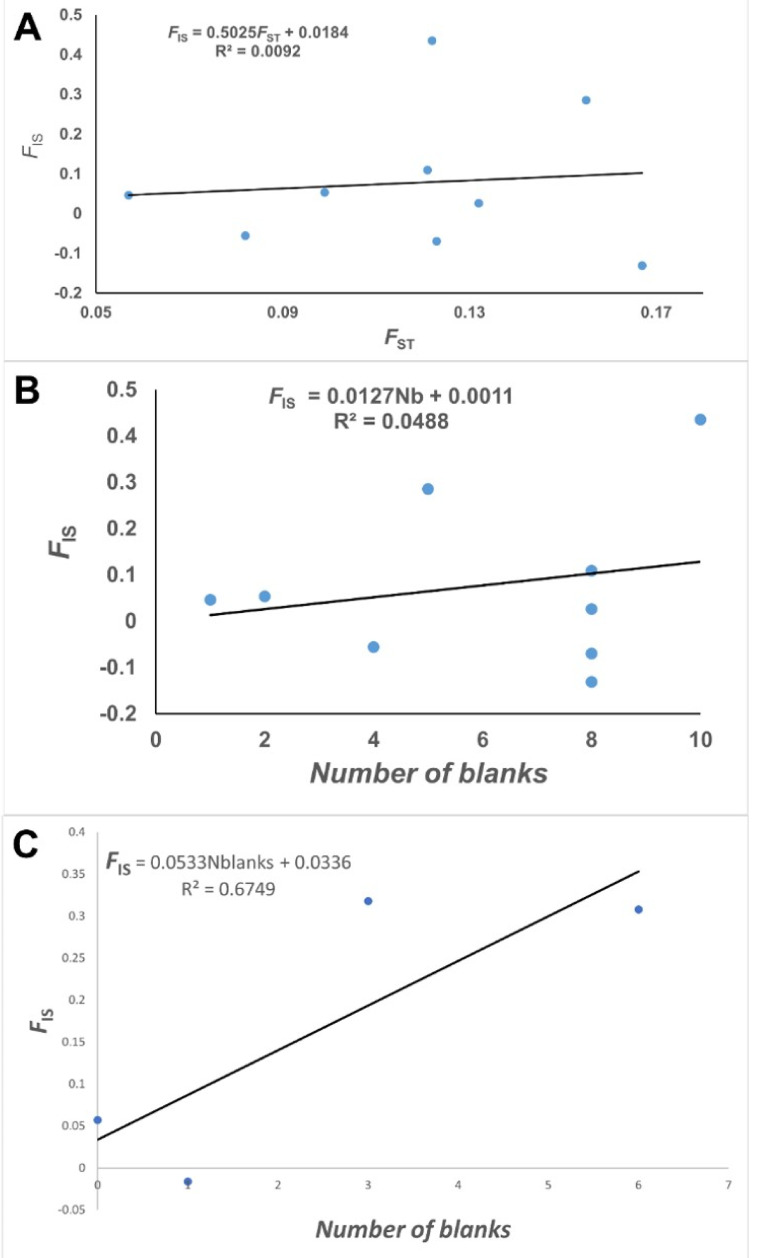
Wright’s *F*_IS_ values *p*-values for panmixia (A) and *F*_ST_ values with *p*-values for subdivision (B) of nine selected loci across field samples only. Dots indicate mean values, dashes show lower and upper limits calculated with the following formula: Li = *F*_XY_ – StdErr × *t* and Ls = *F*_XY_ + StdErr × *t*. Dashes for All loci are the 95% confidence intervals as obtained by jackknife resampling over populations for each locus and by 10,000 bootstraps over loci for the average.

In the field samples only, genetic differentiation remained significant, with *F*_ST_ = 0.067 in 95% CI = [0.043, 0.097] (*p*-value < 0.0001). There was still a significant heterozygote deficit: *F*_IS_ = 0.068 in 95% CI = [−0.02, 0.164] (*p*-value < 0.0002) (Supplementary Figure 6).

**Supplementary Figure 7:**
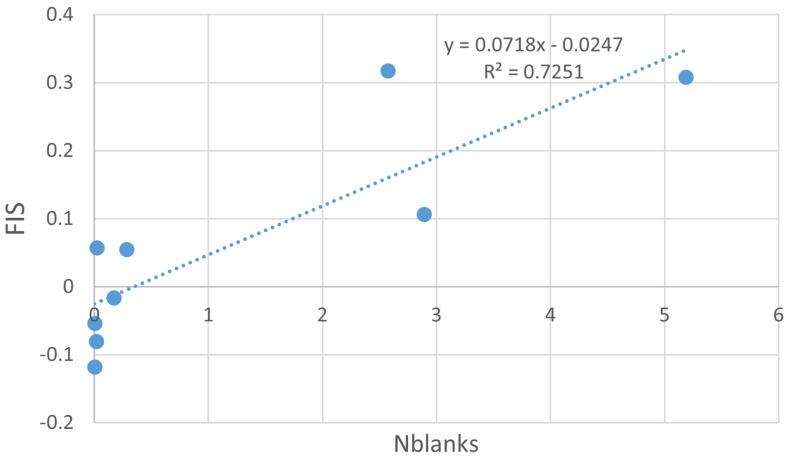
Correlation between *F*_IS_ and FST (A) and between *F*_IS_ and number of blanks (B) for selected nine loci across field samples.

As explained above, null alleles explained most of *F*_IS_ variations. We always observed enough numbers of blanks as compared to the expected ones as provided by FreeNA with the EM algorithm to estimate null allele frequencies (all *p*-values > 0.7). The regression of *F*_IS_ as a function of expected null homozygotes (Nblanks – expected) provided a *R*^2^ = 0.7251 and an intercept of *F*_IS_no-null_ = −0.0247 (Supplementary Figure 7), as expected in a random mating dioecious population [15]. After correction for null alleles with FreeNA and correction for polymorphism using RecodeData, the average genetic differentiation between subsample pairs across the two countries was very high: *F*_ST_FreeNA_′ = 0.3159 in 95% CI = [0.180, 0.457]. Nevertheless, this cannot be translated into an estimate of gene flow since we cannot separate the respective effect of temporal (three generations) and geographic (>150 km) distances.

### Effective population sizes (*N*_e_)

Results of the estimation of effective population sizes are presented in Table 3, where it is indicated that the *F*_IS_ based *N*_e_ = 10, in minmax = [4, 17] is very close to the one obtained with the intercept of the regression *F*_IS_ *~ N*_blanks-expected_ (*N*_e_ = 10.51). This brings confidence on the interpretation regarding the effect of null alleles. The weighted grand average gave effective population sizes: *N*_e_ = 76 in minmax = [16, 192].

**Table 3:** Average effective population size obtained for the *F*_IS_-based, LD-based, Coancestry-based, the one and two locus correlation-based and the sibship frequency-based methods. For each, the minimum and maximum values are given. These were used to compute the grand average, weighted by the number of usable values (Weights) (values different from 0 or infinity).

Methods	Average *N*_e_	*N*_e_min_	*N*_e_max_	Weights
FIS	10	4	17	5
LD	364	54	965	3
Coancestry	29	8	87	4
Correlations	14	14	14	1
Sibship	19	11	24	5
Grand average	76	16	192	

### Genetic differentiation between subsamples within South Africa and Mozambique

Within each country, the average genetic differentiation was small and not significant with *F*_ST_FreeNA_′ = 0.0983 in 95% CI = [−0.0160, 0.2953] (*p*-value = 0.3208) in South Africa and F_ST_FreeNA_′ = −0.0206 in 95% CI = [−0.1344, 0.1036] (*p*-value = 0.8036) on average between traps in Mozambique.

## Discussion

This study aimed to develop novel microsatellite markers for *G. brevipalpis* and to evaluate the suitability and efficiency of these markers to investigate population structure and the level of gene flow between populations in the field. The search for microsatellites across the genome sequence of *G. brevipalpis* indicated that dinucleotide repeats were more common than trinucleotide repeats confirming the theory that the microsatellite abundance decreases with the increase of motif repeat number and repeat length [63]. To our knowledge, this is the first report of a set of microsatellite markers that can be used effectively for *G. brevipalpis,* allowing a broader analysis of the population genetics of this species in southeast Africa.

The selected microsatellite markers used in this study were inspected for their efficiency to explore genetic heterogeneity within and between tested populations using several quality control tests. As a result of the quality control tests, nine markers were retained, with an average genetic diversity *H*_t_ = 0.816, which is a satisfying rate for population genetics studies. Among these nine loci, four (*Gb5*, *Gb35*, *Gb73* and *Gb92*) displayed null alleles at different levels of frequency. As for the *F*_ST_ between the two countries, given the importance of the geographic distances between the two countries, relative to the modest number of generations separating those, it is probable that most of the genetic differentiation observed between the two countries, as measured by *F*_ST_FreeNA_′, was due to geography, though we cannot quantify by what proportion exactly. The same consideration applies to the FCA and NJ-tree analyses. Nevertheless, we can forecast that contemporaneous subsamples from the two zones would lead to a considerable genetic differentiation and thus that these two zones exchange very few immigrants per generation. Regarding effective population sizes, variations of *N*_e_ estimates are often important, with the LD-based estimate giving the highest values and the coancestry-based estimate providing the smallest values [15, 22, 66].

Results of the NJ-tree and FCA indicated that the OVI colony was distant from the wild populations of South Africa and Mozambique. The results agree with the results of de Beer *et al*. [13] who’s morphometric and mitochondrial DNA analysis indicated that the flies from the colony could be clearly distinguished from the field samples of Eswatini, Mozambique and South Africa. However, using the selected microsatellite markers in this study, it was possible to classify the flies collected from Mozambique and South Africa into distinct groups. The difference between our results and those of de Beer *et al*. [13] can be explained by the fact that mtDNA is a conserved region and is not subject to many frequent mutations as compared to microsatellite loci.

The distinction between *G. brevipalpis* collected from different locations in Mozambique and South Africa demonstrates that microsatellite markers will provide an enhanced resolution of the genetic structure of this species and therefore allow accurate investigations of the population genetics, the immigration rate and the dispersal distances in this species. Although these results demonstrate the suitability of the selected loci to explore the genetic diversity of *G. brevipalpis* in the tested locations, the low number of tested samples, and the fact that Mozambique and South Africa populations were investigated at different times did not allow us to calculate dispersal or the immigration rates between these populations. Therefore, the genetic structure of these populations should not be considered finally established. For future studies, it is recommended to analyse more samples collected at the same time from more locations to obtain a complete and conclusive analysis of the population genetics of the flies.

## Conclusions

In this study, nine selected microsatellite markers were characterised and found to be suitable for analysing the population genetics of *G. brevipalpis*. The selected microsatellite markers showed the possibility to differentiate between wild flies from different locations in Mozambique and South Africa as well as the flies from a laboratory population. Analysing more field samples collected from more locations of the same tsetse generation with these microsatellite markers will provide a better understanding of the population genetics and dynamics of *G. brevipalpis*. It will allow for precise assessment of the level of gene flow between adjacent populations that could be targeted with an area-wide integrated pest management strategy.

## Data Availability

Materials described in the paper, including all relevant raw data, are available in this link: https://dataverse.harvard.edu/dataset.xhtml?persistentId=doi:10.7910/DVN/SDRST2. Unpublished sequence data from Otto Koekemoer is available upon reasonable request from the corresponding author.

## Supplementary materials

The Supplementary materials of this article are available at https://www.parasite-journal.org/10.1051/parasite/2023038/olm

*Supplementary File 1*: RMarkdown for statistics in R of the manuscript “Development and characterization of microsatellite markers for the tsetse species *Glossina brevipalpis* and preliminary population genetics analyses”. *Supplementary Table 1*: Selected primers for *Glossina brevipalpis* microsatellite that produce PCR product length of 180–300 nucleotides with minimum 13 repeat of each motif. *Supplementary Table 2*: List of genotypes (alleles) per locus for tsetse fly *Glossina brevipalpis*. Dye-labelled PCR were analysed by fragment analyser and the resulting data were read with Genemapper.
