# Correction Notice to: Development and characterization of microsatellite markers for the tsetse species *Glossina brevipalpis* and preliminary population genetics analyses: Parasite **30**, 34 (2023), https://doi.org/10.1051/parasite/2023038

**DOI:** 10.1051/parasite/2023046

**Published:** 2023-11-15

**Authors:** Fabian Gstöttenmayer, Percy Moyaba, Montse Rodriguez, Fernando C. Mulandane, Hermógenes N. Mucache, Luis Neves, Chantel De Beer, Sophie Ravel, Thierry De Meeûs, Robert L. Mach, Marc J. B. Vreysen, Adly M.M. Abd-Alla

**Affiliations:** 1 Insect Pest Control Laboratory, Joint FAO/IAEA Centre of Nuclear Techniques in Food and Agriculture, Vienna International Centre P.O. Box 100 1400 Vienna Austria; 2 Epidemiology, Parasites and Vectors, Agricultural Research Council-Onderstepoort Veterinary Research 100 Soutpan Road Private Bag X05 Onderstepoort 0110 South Africa; 3 University Eduardo Mondlane, Centro de Biotecnologia Av. de Moçambique Km 1.5 Maputo Mozambique; 4 University of Pretoria, Department of Veterinary Tropical Diseases Private Bag X04 Onderstepoort 0110 South Africa; 5 University of Montpellier, Cirad, IRD, Intertryp Campus International de Baillarguet 34398 Montpellier Cedex 5 France; 6 Institute of Chemical, Environmental, and Bioscience Engineering, Vienna University of Technology Gumpendorfer Straße 1a 1060 Vienna Austria

Regarding this article, published on September 15, 2023, several errors occurred:

Page 6, Table 2:

- The title is incorrect and should be “The 10 polymorphic microsatellite loci selected for *Glossina brevipalpis* including repeat motif, primer sequence, number of alleles (*N*_A_), allele size range, heterozygosity within subsamples (*H*_s_), total heterozygosity (*H*_t_).”
- The artefact “Q8” in the left margin is to be suppressed, as the asterisk after “Gb48” and its reference “X linked”.

Page 7, line 49: A call to Supplementary Figure 7 is missing between *F*_IS_no-null_ = −0.0247 and the comma.

