# Supplementary material for: Development and characterization of microsatellite markers for the tsetse species Glossina brevipalpis and preliminary population genetics analyses
Source: Parasite. 2023 Sep 15;30:34. doi: 10.1051/parasite/2023038 (PMC10503490; doi:10.1051/parasite/2023038)
Supplement: Supplementary file 1 — RMarkdown for statistics in R of the manuscript “Development and characterization of microsatellite markers for the tsetse species Glossina brevipalpis and preliminary population genetics analyses”. [file parasite-30-34-s1.pdf]

Development and characterization of microsatellite markers for the tsetse species Glossina brevipalpis and preliminary population genetics analyses

Fabian Gstöttenmayer1, Percy Moyaba1,2, Montse Rodriguez1, Fernando C. Mulandane3, Hermogenes N. Mucache4, Luis Neves3,4, Chantel De Beer1, Sophie Ravel5, Thierry De Meus5, Philippe Solano5 Robert L. Mach6, Marc J. B. Vreysen1 and Adly M.M. Abd-Alla1*

2023-06-20

```
setwd("C:/Users/GSTOTTENMF/OneDrive - IAEA/Desktop/Manuscript V7/Submission/D
ata_repository")

library(ggplot2)
library(MASS)
library(rmarkdown)
library(knitr)
library(lme4)
library(MuMIn)
library(ggthemes)
library(datasets)
library(plyr)
library(dplyr)
library(tidyverse)
library(coin)

### Analysis of all loci on all samples
### Linkage disequilibrium: BY correction for all loci
p.adjust(c(0.3229,  0.5156, 1,  0.001,  0.6289, 0.8639, 0.7811, 0.8109, 0.874
9, 0.3022, 0.6471, 0.1094, 0.0072, 0.0763, 0.0103, 0.079,  0.9676, 0.2757, 0.
1998, 0.544,  0.5839, 0.0206, 0.3982, 0.2224, 1,  0.9607, 0.3872, 0.1506, 0.4
215, 0.2636, 1,  0.4142, 0.5945, 0.2685, 0.3466, 0.0648, 0.4681, 0.5652, 0.86
01, 0.4104, 0.3353, 0.8572, 0.4667, 0.8822, 0.6735), method = "BY")

##  [1] 1.0000000 1.0000000 1.0000000 0.1977727 1.0000000 1.0000000 1.0000000
##  [8] 1.0000000 1.0000000 1.0000000 1.0000000 1.0000000 0.6790195 1.0000000
## [15] 0.6790195 1.0000000 1.0000000 1.0000000 1.0000000 1.0000000 1.0000000
## [22] 1.0000000 1.0000000 1.0000000 1.0000000 1.0000000 1.0000000 1.0000000
## [29] 1.0000000 1.0000000 1.0000000 1.0000000 1.0000000 1.0000000 1.0000000
## [36] 1.0000000 1.0000000 1.0000000 1.0000000 1.0000000 1.0000000 1.0000000
## [43] 1.0000000 1.0000000 1.0000000

### Correlation between FIS and FST for all loci on all samples
FISFST <- read.csv("FISFST_allloci_allsamples.csv", sep=",", row.names=NULL)
FISFST
```

```
##    Locus   FST    FIS
## 1    Gb5 0.099  0.053
## 2   Gb28 0.121  0.109
## 3   Gb35 0.122  0.435
## 4   Gb48 0.108  0.052
## 5   Gb66 0.057  0.046
## 6   Gb72 0.082 -0.056
## 7   Gb73 0.155  0.285
## 8   Gb92 0.132  0.026
## 9  Gb158 0.123 -0.070
## 10 Gb165 0.167 -0.131
```

```
with(FISFST, cor.test(FST, FIS, alternative="greater", method="spearman"))
```

```
##
##  Spearman's rank correlation rho
##
## data:  FST and FIS
## S = 184, p-value = 0.6336
## alternative hypothesis: true rho is greater than 0
## sample estimates:
##        rho
## -0.1151515
```

```
### Correlation between FIS and number of blanks for all loci on all samples
FISblanksall <- read.csv("FISblanks_allloci_allsamples.csv", sep=",", row.nam
es=NULL)
FISblanksall
```

```
##    Locus Nb    FIS
## 1    Gb5  2  0.053
## 2   Gb28  8  0.109
## 3   Gb35 10  0.435
## 4   Gb48 20  0.052
## 5   Gb66  1  0.046
## 6   Gb72  4 -0.056
## 7   Gb73  5  0.285
## 8   Gb92  8  0.026
## 9  Gb158  8 -0.070
## 10 Gb165  8 -0.131
```

```
with(FISblanksall, cor.test(Nb, FIS, alternative="greater", method="spearman"
))
```

```
## Warning in cor.test.default(Nb, FIS, alternative = "greater", method =
## "spearman"): Cannot compute exact p-value with ties
```

```
##
##  Spearman's rank correlation rho
##
## data:  Nb and FIS
```

```
## S = 146.43, p-value = 0.3784
## alternative hypothesis: true rho is greater than 0
## sample estimates:
##      rho
## 0.112555
```

```r
### Correlation between FIS and number of blanks for 7 loci (Gb5, 28, 35, 66,
73, 92, 165) on all samples
FISblanks7lociall <- read.csv("FISblanks_7loci_allsamples.csv", sep=",", row.
names=NULL)
FISblanks7lociall
```

```
##   Locus Nb    FIS
## 1   Gb5  2  0.053
## 2  Gb28  8  0.109
## 3  Gb35 10  0.435
## 4  Gb66  1  0.046
## 5  Gb73  5  0.285
## 6  Gb92  8  0.026
## 7 Gb165  8 -0.131
```

```r
with(FISblanks7lociall, cor.test(Nb, FIS, alternative="greater", method="spea
rman"))
```

```
## Warning in cor.test.default(Nb, FIS, alternative = "greater", method =
## "spearman"): Cannot compute exact p-value with ties
```

```
##
##  Spearman's rank correlation rho
##
## data:  Nb and FIS
## S = 43.547, p-value = 0.3159
## alternative hypothesis: true rho is greater than 0
## sample estimates:
##       rho
## 0.2223748
```

```r
### Correlation between FIS and number of blanks for 4 loci (Gb 5, 35, 66, 73
) on all samples
FISblanks4lociall <- read.csv("FISblanks_4loci_allsamples.csv", sep=",", row.
names=NULL)
FISblanks4lociall
```

```
##   Locus Nb   FIS
## 1   Gb5  2 0.053
## 2  Gb35 10 0.435
## 3  Gb66  1 0.046
## 4  Gb73  5 0.285
```

```r
with(FISblanks4lociall, cor.test(Nb, FIS, alternative="greater", method="spea
rman"))
```

```
##
##  Spearman's rank correlation rho
##
## data:  Nb and FIS
## S = 0, p-value = 0.04167
## alternative hypothesis: true rho is greater than 0
## sample estimates:
## rho
##   1
```

```
### Short allele dominance
SAD <- read.csv("SAD.csv",sep=",", row.names=NULL)
SAD
```

```
##      loci Allele   Capf p..All.W. Smallf     Weights
## 1    Gb28    227  0.297        NA     NA          NA
## 2    Gb28    228 -0.033        NA     NA          NA
## 3    Gb28    229  0.099        NA     NA          NA
## 4    Gb28    231  0.368        NA     NA          NA
## 5    Gb28    233  0.281        NA     NA          NA
## 6    Gb28    235  0.020        NA     NA          NA
## 7    Gb28    237 -0.004        NA     NA          NA
## 8    Gb28    239  0.010        NA     NA          NA
## 9    Gb28    247  0.478        NA     NA          NA
## 10   Gb28    249 -0.009        NA     NA          NA
## 11   Gb28    257 -0.005        NA     NA          NA
## 12   Gb28    281  0.055        NA     NA          NA
## 13   Gb35    193  1.000     0.014     NA 0.014198783
## 14   Gb35    199  0.413     0.049     NA 0.051524711
## 15   Gb35    205  0.430     0.118     NA 0.133786848
## 16   Gb35    221  0.468     0.153     NA 0.180637544
## 17   Gb35    223  0.027     0.007     NA 0.007049345
## 18   Gb35    227  0.387     0.035     NA 0.036269430
## 19   Gb35    229  0.431     0.326     NA 0.483679525
## 20   Gb35    233  0.643     0.208     NA 0.262626263
## 21   Gb35    235  0.549     0.049     NA 0.051524711
## 22   Gb35    239  0.658     0.042     NA 0.043841336
## 23   Gb48    229 -0.004     0.025 -0.059 0.024375000
## 24   Gb48    251  0.072     0.008 -0.286 0.007936000
## 25   Gb48    253  0.150     0.083  0.091 0.076111000
## 26   Gb48    255  0.183     0.358  0.149 0.229836000
## 27   Gb48    257  0.454     0.217  0.281 0.169911000
## 28   Gb48    259  0.005     0.008 -0.014 0.007936000
## 29   Gb48    261  0.003     0.025 -0.080 0.024375000
## 30   Gb48    264 -0.024     0.042 -0.070 0.040236000
## 31   Gb48    265  0.008     0.008 -0.024 0.007936000
## 32   Gb48    266 -0.096     0.117 -0.212 0.103311000
## 33   Gb48    268 -0.007     0.025 -0.047 0.024375000
## 34   Gb48    269 -0.013     0.017  0.005 0.016711000
## 35   Gb48    277  0.018     0.042 -0.223 0.040236000
```

```
## 36   Gb48    279  0.026      0.025 -0.167 0.024375000
## 37    Gb5    219  0.027      0.006     NA 0.006036217
## 38    Gb5    225  0.266      0.043     NA 0.044932079
## 39    Gb5    227  0.461      0.116     NA 0.131221719
## 40    Gb5    229 -0.080      0.098     NA 0.108647450
## 41    Gb5    231 -0.027      0.037     NA 0.038421599
## 42    Gb5    233  0.265      0.043     NA 0.044932079
## 43    Gb5    235  0.217      0.183     NA 0.223990208
## 44    Gb5    237 -0.019      0.024     NA 0.024590164
## 45    Gb5    239  0.046      0.098     NA 0.108647450
## 46    Gb5    241 -0.030      0.037     NA 0.038421599
## 47    Gb5    243  0.275      0.146     NA 0.170960187
## 48    Gb5    245 -0.117      0.116     NA 0.131221719
## 49    Gb5    247  0.199      0.055     NA 0.058201058
## 50   Gb66    202  0.006      0.006     NA 0.006036217
## 51   Gb66    211 -0.049      0.183     NA 0.223990208
## 52   Gb66    214 -0.007      0.006     NA 0.006036217
## 53   Gb66    220  0.005      0.037     NA 0.038421599
## 54   Gb66    223 -0.005      0.055     NA 0.058201058
## 55   Gb66    226  0.069      0.085     NA 0.092896175
## 56   Gb66    229  0.221      0.049     NA 0.051524711
## 57   Gb66    232  0.155      0.140     NA 0.162790698
## 58   Gb66    235 -0.008      0.043     NA 0.044932079
## 59   Gb66    241  0.078      0.079     NA 0.085776330
## 60   Gb66    244  0.204      0.250     NA 0.333333333
## 61   Gb66    247  0.322      0.037     NA 0.038421599
## 62   Gb66    256  0.003      0.018     NA 0.018329939
## 63   Gb66    259  0.000      0.012     NA 0.012145749
## 64   Gb72    207 -0.030      0.056     NA 0.059322034
## 65   Gb72    211  0.516      0.025     NA 0.025641026
## 66   Gb72    213  0.109      0.075     NA 0.081081081
## 67   Gb72    215 -0.026      0.044     NA 0.046025105
## 68   Gb72    217 -0.024      0.038     NA 0.039501040
## 69   Gb72    219  0.113      0.206     NA 0.259445844
## 70   Gb72    221 -0.067      0.300     NA 0.428571429
## 71   Gb72    223  0.064      0.119     NA 0.135073780
## 72   Gb72    225 -0.039      0.063     NA 0.067235859
## 73   Gb72    227 -0.014      0.019     NA 0.019367992
## 74   Gb72    231 -0.018      0.031     NA 0.031991744
## 75   Gb72    235 -0.008      0.006     NA 0.006036217
## 76   Gb72    239 -0.005      0.013     NA 0.013171226
## 77   Gb72    245  0.008      0.006     NA 0.006036217
## 78  Gb158    187 -0.075      0.086     NA 0.094091904
## 79  Gb158    191  0.012      0.026     NA 0.026694045
## 80  Gb158    193  0.115      0.336     NA 0.506024096
## 81  Gb158    195  0.070      0.257     NA 0.345895020
## 82  Gb158    197 -0.025      0.132     NA 0.152073733
## 83  Gb158    199  0.243      0.099     NA 0.109877913
## 84  Gb158    201  0.027      0.007     NA 0.007049345
## 85  Gb158    205 -0.056      0.059     NA 0.062699256
```

```
## 86  Gb165    210 -0.152     0.547     NA 1.207505519
## 87  Gb165    212  0.280     0.267     NA 0.364256480
## 88  Gb165    214  0.269     0.087     NA 0.095290252
## 89  Gb165    218 -0.007     0.007     NA 0.007049345
## 90  Gb165    220 -0.066     0.093     NA 0.102535832
## 91   Gb73    228  0.369     0.095     NA 0.104972376
## 92   Gb73    230  0.132     0.070     NA 0.075268817
## 93   Gb73    238 -0.010     0.013     NA 0.013171226
## 94   Gb73    240  0.806     0.089     NA 0.097694841
## 95   Gb73    242  0.495     0.095     NA 0.104972376
## 96   Gb73    244 -0.044     0.051     NA 0.053740780
## 97   Gb73    246  0.396     0.089     NA 0.097694841
## 98   Gb73    248  0.543     0.247     NA 0.328021248
## 99   Gb73    250  0.334     0.228     NA 0.295336788
## 100  Gb73    254  0.005     0.013     NA 0.013171226
## 101  Gb73    256 -0.001     0.013     NA 0.013171226
## 102  Gb92    278 -0.011        NA     NA          NA
## 103  Gb92    282  0.330        NA     NA          NA
## 104  Gb92    284  0.005        NA     NA          NA
## 105  Gb92    286 -0.005        NA     NA          NA
## 106  Gb92    290 -0.045        NA     NA          NA
## 107  Gb92    292  0.326        NA     NA          NA
## 108  Gb92    294  0.073        NA     NA          NA
## 109  Gb92    300 -0.018        NA     NA          NA
## 110  Gb92    302  0.069        NA     NA          NA
## 111  Gb92    304  0.256        NA     NA          NA
## 112  Gb92    306  0.013        NA     NA          NA
## 113  Gb92    314 -0.010        NA     NA          NA

SAD_Gb28 <- subset(SAD,loci=="Gb28")
SAD_Gb28

##    loci Allele   Capf p..All.W. Smallf Weights
## 1  Gb28    227  0.297        NA     NA      NA
## 2  Gb28    228 -0.033        NA     NA      NA
## 3  Gb28    229  0.099        NA     NA      NA
## 4  Gb28    231  0.368        NA     NA      NA
## 5  Gb28    233  0.281        NA     NA      NA
## 6  Gb28    235  0.020        NA     NA      NA
## 7  Gb28    237 -0.004        NA     NA      NA
## 8  Gb28    239  0.010        NA     NA      NA
## 9  Gb28    247  0.478        NA     NA      NA
## 10 Gb28    249 -0.009        NA     NA      NA
## 11 Gb28    257 -0.005        NA     NA      NA
## 12 Gb28    281  0.055        NA     NA      NA

with(SAD_Gb28, cor.test(Allele, Capf, alternative="less", method="spearman"))

##
##  Spearman's rank correlation rho
##
```

```
## data:  Allele and Capf
## S = 352, p-value = 0.2355
## alternative hypothesis: true rho is less than 0
## sample estimates:
##        rho
## -0.2307692
```

```
SAD_Gb35 <- subset(SAD,loci=="Gb35")
SAD_Gb35
```

```
##    loci Allele  Capf p..All.W. Smallf     Weights
## 13 Gb35    193 1.000     0.014     NA 0.014198783
## 14 Gb35    199 0.413     0.049     NA 0.051524711
## 15 Gb35    205 0.430     0.118     NA 0.133786848
## 16 Gb35    221 0.468     0.153     NA 0.180637544
## 17 Gb35    223 0.027     0.007     NA 0.007049345
## 18 Gb35    227 0.387     0.035     NA 0.036269430
## 19 Gb35    229 0.431     0.326     NA 0.483679525
## 20 Gb35    233 0.643     0.208     NA 0.262626263
## 21 Gb35    235 0.549     0.049     NA 0.051524711
## 22 Gb35    239 0.658     0.042     NA 0.043841336
```

```
with(SAD_Gb35, cor.test(Allele, Capf, alternative="less", method="spearman"))
```

```
##
##  Spearman's rank correlation rho
##
## data:  Allele and Capf
## S = 128, p-value = 0.7431
## alternative hypothesis: true rho is less than 0
## sample estimates:
##       rho
## 0.2242424
```

```
SAD_Gb48 <- subset(SAD,loci=="Gb48")
SAD_Gb48
```

```
##    loci Allele   Capf p..All.W. Smallf  Weights
## 23 Gb48    229 -0.004     0.025 -0.059 0.024375
## 24 Gb48    251  0.072     0.008 -0.286 0.007936
## 25 Gb48    253  0.150     0.083  0.091 0.076111
## 26 Gb48    255  0.183     0.358  0.149 0.229836
## 27 Gb48    257  0.454     0.217  0.281 0.169911
## 28 Gb48    259  0.005     0.008 -0.014 0.007936
## 29 Gb48    261  0.003     0.025 -0.080 0.024375
## 30 Gb48    264 -0.024     0.042 -0.070 0.040236
## 31 Gb48    265  0.008     0.008 -0.024 0.007936
## 32 Gb48    266 -0.096     0.117 -0.212 0.103311
## 33 Gb48    268 -0.007     0.025 -0.047 0.024375
## 34 Gb48    269 -0.013     0.017  0.005 0.016711
```

```
## 35 Gb48    277  0.018     0.042 -0.223 0.040236
## 36 Gb48    279  0.026     0.025 -0.167 0.024375

with(SAD_Gb48, cor.test(Allele, Capf, alternative="less", method="spearman"))

##
##  Spearman's rank correlation rho
##
## data:  Allele and Capf
## S = 622, p-value = 0.09867
## alternative hypothesis: true rho is less than 0
## sample estimates:
##       rho
## -0.367033

with(SAD_Gb48, cor.test(Smallf, Weights, alternative="less", method="spearman
"))

## Warning in cor.test.default(Smallf, Weights, alternative = "less", method
=
## "spearman"): Cannot compute exact p-value with ties

##
##  Spearman's rank correlation rho
##
## data:  Smallf and Weights
## S = 331.95, p-value = 0.8251
## alternative hypothesis: true rho is less than 0
## sample estimates:
##      rho
## 0.270429

lm_SAD_Gb48 <- lm(Smallf ~ Allele, data=SAD_Gb48, Weights=weight)

## Warning: In lm.fit(x, y, offset = offset, singular.ok = singular.ok, ...)
:
##  extra argument 'Weights' will be disregarded

summary(lm_SAD_Gb48)

##
## Call:
## lm(formula = Smallf ~ Allele, data = SAD_Gb48, Weights = weight)
##
## Residuals:
##       Min        1Q    Median        3Q       Max
## -0.270353 -0.100216  0.004299  0.066836  0.315508
##
## Coefficients:
##              Estimate Std. Error t value Pr(>|t|)
## (Intercept)  0.773365   0.897041   0.862    0.406
## Allele      -0.003143   0.003434  -0.915    0.378
```

```
##
## Residual standard error: 0.1531 on 12 degrees of freedom
## Multiple R-squared:  0.06526,    Adjusted R-squared:  -0.01263
## F-statistic: 0.8378 on 1 and 12 DF,  p-value: 0.3781
```

```
SAD_Gb5 <- subset(SAD,loci=="Gb5")
SAD_Gb5
```

```
##    loci Allele   Capf p..All.W. Smallf     Weights
## 37  Gb5    219  0.027     0.006     NA 0.006036217
## 38  Gb5    225  0.266     0.043     NA 0.044932079
## 39  Gb5    227  0.461     0.116     NA 0.131221719
## 40  Gb5    229 -0.080     0.098     NA 0.108647450
## 41  Gb5    231 -0.027     0.037     NA 0.038421599
## 42  Gb5    233  0.265     0.043     NA 0.044932079
## 43  Gb5    235  0.217     0.183     NA 0.223990208
## 44  Gb5    237 -0.019     0.024     NA 0.024590164
## 45  Gb5    239  0.046     0.098     NA 0.108647450
## 46  Gb5    241 -0.030     0.037     NA 0.038421599
## 47  Gb5    243  0.275     0.146     NA 0.170960187
## 48  Gb5    245 -0.117     0.116     NA 0.131221719
## 49  Gb5    247  0.199     0.055     NA 0.058201058
```

```
with(SAD_Gb5, cor.test(Allele, Capf, alternative="less", method="spearman"))
```

```
##
##  Spearman's rank correlation rho
##
## data:  Allele and Capf
## S = 440, p-value = 0.2467
## alternative hypothesis: true rho is less than 0
## sample estimates:
##        rho
## -0.2087912
```

```
SAD_Gb66 <- subset(SAD,loci=="Gb66")
SAD_Gb66
```

```
##    loci Allele   Capf p..All.W. Smallf     Weights
## 50 Gb66    202  0.006     0.006     NA 0.006036217
## 51 Gb66    211 -0.049     0.183     NA 0.223990208
## 52 Gb66    214 -0.007     0.006     NA 0.006036217
## 53 Gb66    220  0.005     0.037     NA 0.038421599
## 54 Gb66    223 -0.005     0.055     NA 0.058201058
## 55 Gb66    226  0.069     0.085     NA 0.092896175
## 56 Gb66    229  0.221     0.049     NA 0.051524711
## 57 Gb66    232  0.155     0.140     NA 0.162790698
## 58 Gb66    235 -0.008     0.043     NA 0.044932079
## 59 Gb66    241  0.078     0.079     NA 0.085776330
## 60 Gb66    244  0.204     0.250     NA 0.333333333
## 61 Gb66    247  0.322     0.037     NA 0.038421599
```

```
## 62 Gb66    256  0.003     0.018     NA 0.018329939
## 63 Gb66    259  0.000     0.012     NA 0.012145749
```

```
with(SAD_Gb66, cor.test(Allele, Capf, alternative="less", method="spearman"))
```

```
## 
##  Spearman's rank correlation rho
## 
## data:  Allele and Capf
## S = 298, p-value = 0.8895
## alternative hypothesis: true rho is less than 0
## sample estimates:
##       rho 
## 0.3450549
```

```
SAD_Gb72 <- subset(SAD,loci=="Gb72")
SAD_Gb72
```

```
##    loci Allele   Capf p..All.W. Smallf     Weights
## 64 Gb72    207 -0.030     0.056     NA 0.059322034
## 65 Gb72    211  0.516     0.025     NA 0.025641026
## 66 Gb72    213  0.109     0.075     NA 0.081081081
## 67 Gb72    215 -0.026     0.044     NA 0.046025105
## 68 Gb72    217 -0.024     0.038     NA 0.039501040
## 69 Gb72    219  0.113     0.206     NA 0.259445844
## 70 Gb72    221 -0.067     0.300     NA 0.428571429
## 71 Gb72    223  0.064     0.119     NA 0.135073780
## 72 Gb72    225 -0.039     0.063     NA 0.067235859
## 73 Gb72    227 -0.014     0.019     NA 0.019367992
## 74 Gb72    231 -0.018     0.031     NA 0.031991744
## 75 Gb72    235 -0.008     0.006     NA 0.006036217
## 76 Gb72    239 -0.005     0.013     NA 0.013171226
## 77 Gb72    245  0.008     0.006     NA 0.006036217
```

```
with(SAD_Gb72, cor.test(Allele, Capf, alternative="less", method="spearman"))
```

```
## 
##  Spearman's rank correlation rho
## 
## data:  Allele and Capf
## S = 454, p-value = 0.506
## alternative hypothesis: true rho is less than 0
## sample estimates:
##         rho 
## 0.002197802
```

```
SAD_Gb158 <- subset(SAD,loci=="Gb158")
SAD_Gb158
```

```
##     loci Allele   Capf p..All.W. Smallf     Weights
## 78 Gb158    187 -0.075     0.086     NA 0.094091904
## 79 Gb158    191  0.012     0.026     NA 0.026694045
```

```
## 80 Gb158    193  0.115     0.336     NA 0.506024096
## 81 Gb158    195  0.070     0.257     NA 0.345895020
## 82 Gb158    197 -0.025     0.132     NA 0.152073733
## 83 Gb158    199  0.243     0.099     NA 0.109877913
## 84 Gb158    201  0.027     0.007     NA 0.007049345
## 85 Gb158    205 -0.056     0.059     NA 0.062699256
```

```
with(SAD_Gb158, cor.test(Allele, Capf, alternative="less", method="spearman")
)
```

```
##
##  Spearman's rank correlation rho
##
## data:  Allele and Capf
## S = 72, p-value = 0.6483
## alternative hypothesis: true rho is less than 0
## sample estimates:
##       rho
## 0.1428571
```

```
SAD_Gb73 <- subset(SAD,loci=="Gb73")
SAD_Gb73
```

```
##     loci Allele   Capf p..All.W. Smallf    Weights
## 91  Gb73    228  0.369     0.095     NA 0.10497238
## 92  Gb73    230  0.132     0.070     NA 0.07526882
## 93  Gb73    238 -0.010     0.013     NA 0.01317123
## 94  Gb73    240  0.806     0.089     NA 0.09769484
## 95  Gb73    242  0.495     0.095     NA 0.10497238
## 96  Gb73    244 -0.044     0.051     NA 0.05374078
## 97  Gb73    246  0.396     0.089     NA 0.09769484
## 98  Gb73    248  0.543     0.247     NA 0.32802125
## 99  Gb73    250  0.334     0.228     NA 0.29533679
## 100 Gb73    254  0.005     0.013     NA 0.01317123
## 101 Gb73    256 -0.001     0.013     NA 0.01317123
```

```
with(SAD_Gb73, cor.test(Allele, Capf, alternative="less", method="spearman"))
```

```
##
##  Spearman's rank correlation rho
##
## data:  Allele and Capf
## S = 250, p-value = 0.3468
## alternative hypothesis: true rho is less than 0
## sample estimates:
##        rho
## -0.1363636
```

```
SAD_Gb92 <- subset(SAD,loci=="Gb92")
SAD_Gb92
```

```
##     loci Allele   Capf p..All.W. Smallf Weights
## 102 Gb92    278 -0.011        NA     NA      NA
## 103 Gb92    282  0.330        NA     NA      NA
## 104 Gb92    284  0.005        NA     NA      NA
## 105 Gb92    286 -0.005        NA     NA      NA
## 106 Gb92    290 -0.045        NA     NA      NA
## 107 Gb92    292  0.326        NA     NA      NA
## 108 Gb92    294  0.073        NA     NA      NA
## 109 Gb92    300 -0.018        NA     NA      NA
## 110 Gb92    302  0.069        NA     NA      NA
## 111 Gb92    304  0.256        NA     NA      NA
## 112 Gb92    306  0.013        NA     NA      NA
## 113 Gb92    314 -0.010        NA     NA      NA
```

```
with(SAD_Gb92, cor.test(Allele, Capf, alternative="less", method="spearman"))
```

```
##
##  Spearman's rank correlation rho
##
## data:  Allele and Capf
## S = 276, p-value = 0.5481
## alternative hypothesis: true rho is less than 0
## sample estimates:
##        rho
## 0.03496503
```

```
SAD_Gb165 <- subset(SAD,loci=="Gb165")
SAD_Gb165
```

```
##     loci Allele   Capf p..All.W. Smallf     Weights
## 86 Gb165    210 -0.152     0.547     NA 1.207505519
## 87 Gb165    212  0.280     0.267     NA 0.364256480
## 88 Gb165    214  0.269     0.087     NA 0.095290252
## 89 Gb165    218 -0.007     0.007     NA 0.007049345
## 90 Gb165    220 -0.066     0.093     NA 0.102535832
```

```
with(SAD_Gb165, cor.test(Allele, Capf, alternative="less", method="spearman")
)
```

```
##
##  Spearman's rank correlation rho
##
## data:  Allele and Capf
## S = 20, p-value = 0.525
## alternative hypothesis: true rho is less than 0
## sample estimates:
## rho
##   0
```

```
### Linkage disequilibrium: BY correction for 9 selected loci
p.adjust(c(0.3265,  0.5193, 0.0006, 0.6329, 0.864,  0.7794, 0.821,  0.8735, 0
```

```
.2963, 0.1097, 0.0085, 0.0715, 0.0115,
           0.081,   0.9701, 0.2031, 0.5426, 0.5946, 0.0201, 0.4044, 0.2177, 1
,  0.3921, 0.5911, 0.269,  0.3584, 0.0654,
           0.4602,  0.5638, 0.8668, 0.4043, 0.3298, 0.8541, 0.4636, 0.8868, 0
.6678), method="BY")
```

```
##  [1] 1.00000000 1.00000000 0.09017048 1.00000000 1.00000000 1.00000000
##  [7] 1.00000000 1.00000000 1.00000000 1.00000000 0.57608917 1.00000000
## [13] 0.57608917 1.00000000 1.00000000 1.00000000 1.00000000 1.00000000
## [19] 0.75517776 1.00000000 1.00000000 1.00000000 1.00000000 1.00000000
## [25] 1.00000000 1.00000000 1.00000000 1.00000000 1.00000000 1.00000000
## [31] 1.00000000 1.00000000 1.00000000 1.00000000 1.00000000 1.00000000
```

```
### Correlation between FIS and FST for 9 selected loci on all samples
FISFST9loci <- read.csv("FISFST_9loci_allsamples.csv", sep=",", row.names=NUL
L)
FISFST9loci
```

```
##    Locus   FST    FIS
## 1    Gb5 0.099  0.053
## 2   Gb28 0.121  0.109
## 3   Gb35 0.122  0.435
## 4   Gb66 0.057  0.046
## 5   Gb72 0.082 -0.056
## 6   Gb73 0.155  0.285
## 7   Gb92 0.132  0.026
## 8  Gb158 0.123 -0.070
## 9  Gb165 0.167 -0.131
## 10   All 0.116  0.082
```

```
with(FISFST9loci, cor.test(FST, FIS, alternative="greater", method="spearman"
))
```

```
## 
##  Spearman's rank correlation rho
## 
## data:  FST and FIS
## S = 182, p-value = 0.6206
## alternative hypothesis: true rho is greater than 0
## sample estimates:
##        rho 
## -0.1030303
```

```
### Correlation between FIS and FST for 9 selected loci on field samples only
FISFST9locifieldsamples <- read.csv("FISFST_9loci_fieldsamples.csv", sep=",",
row.names=NULL)
FISFST9locifieldsamples
```

```
##   Locus   FST    FIS
## 1   Gb5 0.056  0.057
## 2  Gb28 0.079  0.055
```

```
## 3  Gb35 0.171  0.308
## 4  Gb66 0.040 -0.016
## 5  Gb72 0.058 -0.054
## 6  Gb73 0.080  0.318
## 7  Gb92 0.007  0.107
## 8 Gb158 0.022 -0.080
## 9 Gb165 0.103 -0.118
```

```
with(FISFST9locifieldsamples, cor.test(FST, FIS, alternative="greater", metho
d="spearman"))
```

```
##
##  Spearman's rank correlation rho
##
## data:  FST and FIS
## S = 100, p-value = 0.3389
## alternative hypothesis: true rho is greater than 0
## sample estimates:
##       rho
## 0.1666667
```

```
### Correlation between FIS and number of blanks for selected 9 loci on field
samples
FISblanks9loci_fieldsamples <- read.csv("FISblanks_9loci_fieldsamples.csv", s
ep=",", row.names=NULL)
FISblanks9loci_fieldsamples
```

```
##   Locus Nb    FIS
## 1   Gb5  0  0.057
## 2  Gb28  8  0.055
## 3  Gb35  6  0.308
## 4  Gb66  1 -0.016
## 5  Gb72  3 -0.054
## 6  Gb73  3  0.318
## 7  Gb92  5  0.107
## 8 Gb158  4 -0.080
## 9 Gb165  4 -0.118
```

```
with(FISblanks9loci_fieldsamples, cor.test(Nb, FIS, alternative="greater", me
thod="spearman"))
```

```
## Warning in cor.test.default(Nb, FIS, alternative = "greater", method =
## "spearman"): Cannot compute exact p-value with ties
```

```
##
##  Spearman's rank correlation rho
##
## data:  Nb and FIS
## S = 108.91, p-value = 0.4065
## alternative hypothesis: true rho is greater than 0
## sample estimates:
```

```
##        rho
## 0.09244024
```

```r
### Correlation between FIS and number of blanks for loci without null allele
s (Gb5, 35, 66, 73, 92)
FISblankslocinonulls_fieldsamples <- read.csv("FISblanks_locinonulls_fieldsam
ples.csv", sep=",", row.names=NULL)
FISblankslocinonulls_fieldsamples
```

```
##   Locus Nb FIS_nulls
## 1   Gb5  0     0.057
## 2  Gb28  8        NA
## 3  Gb35  6     0.308
## 4  Gb66  1    -0.016
## 5  Gb72  3        NA
## 6  Gb73  3     0.318
## 7  Gb92  5     0.107
## 8 Gb158  4        NA
## 9 Gb165  4        NA
```

```r
with(FISblankslocinonulls_fieldsamples, cor.test(Nb, FIS_nulls, alternative="
greater", method="spearman"))
```

```
## 
##  Spearman's rank correlation rho
## 
## data:  Nb and FIS_nulls
## S = 8, p-value = 0.175
## alternative hypothesis: true rho is greater than 0
## sample estimates:
## rho 
## 0.6
```
